# Psoriasis: microbiome dysbiosis and pathogenic mechanisms

**DOI:** 10.3389/fimmu.2026.1714515

**Published:** 2026-03-06

**Authors:** Binghao Wang, Yi Zhang, Li Lin, Songyan Wang, Suqing Yang

**Affiliations:** 1 First Clinical Medical College, Heilongjiang University of Chinese Medicine, Harbin, China; 2 Department of Dermatology, First Affiliated Hospital of Heilongjiang University of Chinese Medicine, Harbin, Heilongjiang, China

**Keywords:** causality, gut-skin axis, IL-23/Th17 pathway, microbiome dysbiosis, pathogenic mechanism, psoriasis, therapeutic modulation

## Abstract

Psoriasis is a chronic immune-mediated inflammatory disease whose pathogenesis is a triad of genetic predisposition, immune dysregulation, and environmental triggers. This review provides a novel, in-depth synthesis arguing that microbial dysbiosis is not merely an associative phenomenon but a central regulatory node within this triad, actively shaping immune responses and clinical phenotypes. We move beyond cataloging microbial shifts to construct a detailed mechanistic framework of the gut-skin axis. Gut dysbiosis; characterized by reduced diversity, a diminished Bacteroidetes/Firmicutes ratio, and depleted SCFA producers, compromises intestinal barrier integrity, reduces systemic immunoregulatory tone via diminished SCFA signaling, and promotes Th17 polarization. This systemic inflammation is directly communicated to the skin. Concurrently, cutaneous dysbiosis, featuring *Staphylococcus aureus* dominance and fungal alterations, disrupts the local barrier, provides chronic antigenic stimulation, and amplifies IL-17-driven inflammation, creating a self-sustaining loop. Crucially, we analyze how specific infections (*HCV, H. pylori, Streptococcus*) act as environmental triggers by sharing or activating these very pathways. The bidirectional relationship with therapy is dissected: while biologics induce drug-specific microbiome shifts that often correlate with clinical normalization, they also carry infection risks that must be strategically managed. Emerging microbiome-targeted interventions like specific probiotics show promise but are hampered by methodological inconsistencies. This review uniquely highlights the causality gap and proposes that future breakthroughs require a shift from correlation to mechanism. We conclude that the microbiome is a dynamic interface between genes and environment in psoriasis; its successful integration into diagnostic and therapeutic paradigms demands standardized multi-omics approaches, functional validation, and personalized medicine strategies that target this critical axis.

## 1 Introduction

Psoriasis is a chronic, immune-mediated systemic disorder with primary cutaneous manifestations, whose pathogenesis represents a complex interplay between a susceptible genetic background, dysregulated immune responses, and environmental exposures (1, 2). While the centrality of the IL-23/Th17 axis is well-established, the specific environmental factors that initiate and perpetuate this dysregulation remain a critical frontier. This review posits that the microbiome; the collective communities of commensal, symbiotic, and pathogenic microorganisms residing on barrier surfaces, is a principal environmental modulator and an integral component of the disease mechanism itself, rather than a passive bystander (3–5).

The conceptualization of psoriasis has evolved from a purely keratinocyte-centric disorder to a systemic immune disease. The identification of the IL-23/Th17 axis as a central driver represented a paradigm shift, leading to the development of highly effective targeted biologics (6, 7). However, a critical unanswered question persists: what initiates this axis in genetically predisposed individuals? Environmental factors are widely implicated, but their precise mechanisms have been elusive. Among these, infectious triggers like streptococcal pharyngitis preceding guttate psoriasis have long been recognized, hinting at a microbial role (8). The advent of high-throughput sequencing has revolutionized our ability to profile microbial communities, revealing that psoriasis patients harbor distinct microbial ecosystems, or dysbiosis, both in the gut and on the skin compared to healthy individuals (9–12). These findings have ignited interest in the “gut-skin axis,” a theoretical framework proposing bidirectional communication between these two barrier organs via metabolic, neural, and immune pathways (5).

However, the field is currently saturated with descriptive studies cataloging taxonomic changes. The unique contribution and depth of this review lie in its attempt to weave these observations into a cohesive, mechanistic narrative. We propose a unified “Gut-Skin-Immune Axis” model where dysbiosis is an upstream event that: (1) compromises barrier function at multiple levels, leading to increased translocation of microbial products; (2) directly modulates systemic and local immune cell differentiation, critically skewing the Th17/Treg balance towards inflammation; (3) generates pro-inflammatory microbial metabolites while depleting anti-inflammatory ones (SCFAs), thereby altering the host’s metabolic and immune landscape; and (4) can be directly triggered by specific pathogens, thereby linking classic infectious triggers to chronic inflammatory pathways via shared immune mechanisms (13–15). This model moves beyond association to propose testable hypotheses about causality.

**Figure 1:**
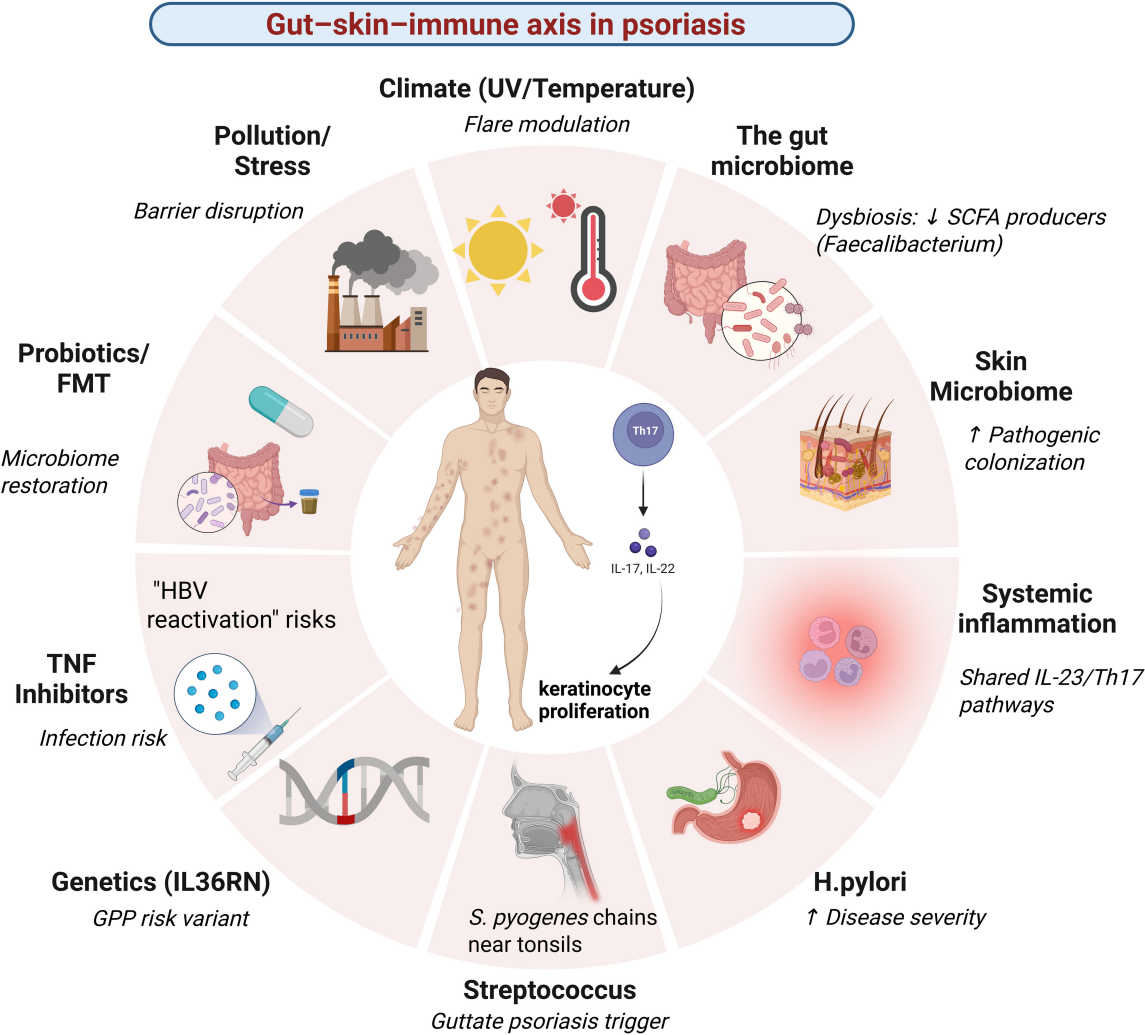
Conceptual framework of the gut–skin–immune axis in psoriasis. Psoriasis is a systemic immune-mediated disease arising from the interaction between genetic susceptibility, environmental exposures, and dysregulated host–microbiome interactions. Alterations in the gut and skin microbiome act as upstream modulators by impairing epithelial barrier integrity, promoting translocation of microbial products, and shaping systemic immune responses. These processes converge on shared IL-23/Th17 inflammatory pathways, leading to immune cell trafficking to the skin, keratinocyte hyperproliferation, and chronic inflammation. Environmental factors, microbial perturbations, systemic inflammatory states, and therapeutic interventions dynamically modulate this interconnected gut–skin–immune network.

This review systematically synthesizes evidence to address the core mechanistic question: How do alterations in microbial communities translate into the hyperproliferative, inflammatory phenotype of psoriasis? We critically evaluate not just the “what” of dysbiosis (taxonomic changes), but the “so what” (functional consequences and immune crosstalk). Furthermore, we analyze the bidirectional relationship with therapies, exploring how treatments alter the microbiome and, conversely, how the microbiome may influence treatment response (16–18). This reverse-engineering approach provides compelling, albeit indirect, evidence for the microbiome’s functional role. Finally, we confront the major limitations head-on-methodological heterogeneity, the profound challenge of proving causality in human studies, and the neglect of non-bacterial kingdoms like fungi and viruses, and provide a targeted roadmap for future research aimed at moving from associative links to actionable, microbiome-informed diagnostics and therapeutics (19–21). The following sections deconstruct this axis, beginning with its foundational role in core pathophysiology, to build a comprehensive picture of psoriasis as a disease of barrier dysfunction and dysregulated host-microbe dialogue **Figure 1**.

## 2 Pathogenicity, pathophysiology, and biological mechanisms

Psoriasis pathogenesis is a cascade where genetic risk sets the stage, environmental triggers pull the curtain, and immune dysregulation drives the plot. The microbiome acts as a key environmental trigger and a continuous modulator of the immune response, serving as the dynamic interface between an individual’s genome and their environment. This section delves into the intricate biological mechanisms, expanding on how microbial communities influence and are influenced by each component of the pathogenic triad.

### 2.1 Core immune dysregulation: the IL-23/Th17 axis as the final common pathway

The IL-23/Th17 axis is the cornerstone of psoriatic inflammation. Dendritic cells and macrophages in the dermis produce IL-23, a cytokine that is critical for the expansion, survival, and pathogenicity of a subset of T helper cells called Th17 cells (7, 22). IL-23 stabilizes the Th17 phenotype by sustaining the expression of the lineage-defining transcription factor RORγt. These pathogenic Th17 cells then secrete a characteristic set of effector cytokines; primarily IL-17A, IL-17F, IL-22, and TNF-α, that directly target keratinocytes, the primary cellular component of the epidermis (6, 23). IL-17A/F signaling is particularly potent, acting through the IL-17 receptor complex on keratinocytes. This engagement activates a downstream signaling cascade involving the adaptor protein Act1, leading to the induction of the key transcription factor IκBζ (encoded by *NFKBIZ*) (24). IκBζ acts as a master regulator for a suite of psoriasis-related genes. It does not bind DNA directly but associates with NF-κB p50 subunits to drive the expression of antimicrobial peptides (DEFB4, S100A7/8/9) and chemokines (CXCL1, CXCL8) that are crucial for recruiting neutrophils, a hallmark of psoriatic plaques (24, 25). The rapid and profound clinical efficacy of anti-IL-17A biologics like secukinumab and ixekizumab, which dramatically reduce IκBζ expression in lesions, provides the most compelling human evidence for this pathway’s centrality (26). TNF-α, often produced by the same Th17 cells and other immune cells, synergizes powerfully with IL-17, amplifying the inflammatory cascade, enhancing endothelial activation to promote vascular changes, and further driving keratinocyte proliferation and cytokine production (27, 28). This synergistic cytokine milieu creates a self-amplifying inflammatory circuit that is central to plaque maintenance.

### 2.2 Keratinocyte autoinflammation

Keratinocytes are not passive targets but active immunocompetent cells that significantly contribute to psoriatic inflammation. Upon stimulation by IL-17, TNF-α, or direct microbial products (via TLRs), they produce their own array of cytokines, chemokines, and antimicrobial peptides, creating potent autocrine and paracrine loops that sustain inflammation independently of continuous immune cell input (29, 30). A critical and underappreciated loop involves IL-33 and its receptor ST2 (IL1RL1), which are constitutively expressed by keratinocytes. Psoriasis-relevant stimuli, including IL-17 itself, trigger the release of bioactive IL-33 from keratinocytes (31). This cytokine then acts back on the same or neighboring keratinocytes via the ST2 receptor, inducing a robust pro-inflammatory gene program (*IL6*, *CXCL8*, *CCL20*) that powerfully synergizes with signals from the Th17 axis (31, 32). This IL-33/ST2 autocrine loop represents a therapeutically exploitable, keratinocyte-intrinsic amplifier of inflammation. It may explain the residual inflammation seen in some patients on anti-IL-17 therapy and presents a novel target for intervention, with anti-IL-33 biologics currently under investigation (33). Furthermore, keratinocytes contribute to innate immune activation by expressing pattern recognition receptors (PRRs) that sense dysbiotic microbiota, leading to the production of IL-1β, IL-6, and IL-23, thus potentially initiating the inflammatory cascade from the epithelial side (34).

### 2.3 Genetic predisposition: setting the stage

Specific genetic variants strongly influence susceptibility, phenotype, and treatment response in psoriasis. The strongest genetic signal lies within the Major Histocompatibility Complex (MHC). HLA-C*06:02 is the classic allele associated with early-onset, type I psoriasis and guttate psoriasis, which is often triggered by streptococcal infection (35). Other alleles like HLA-B*46:01 are linked to different clinical patterns, suggesting that HLA molecules may shape disease by presenting distinct microbial or autoantigens to T cells (35). Non-HLA genes also contribute significantly. Gain-of-function mutations in *CARD14*, a regulator of NF-κB signaling, lead to a rare, severe form of pustular psoriasis, directly linking innate immune signaling in keratinocytes to disease (36). Variants in genes encoding IL-23 receptor (*IL23R*) and components of the IL-23 signaling pathway further underscore the importance of this axis (37). Notably, the microbiome can influence the expression of such genetic predispositions, acting as a key environmental factor that determines whether genetic risk translates into clinical disease. This concept, known as gene-environment interaction, suggests that dysbiosis may lower the threshold for immune activation in genetically susceptible hosts, for example, by providing antigens that cross-react with self-proteins (molecular mimicry) or by disrupting immune regulatory networks that would otherwise keep autoreactive cells in check (38, 39).

### 2.4 The microbiome and gut-skin axis: a mechanistic deep dive

This section provides the detailed, analytical narrative called for by reviewers, moving beyond mere description to propose a causal framework linking gut dysbiosis to systemic and cutaneous inflammation.

#### 2.4.1 Gut dysbiosis as an instigator of systemic immune bias

Multiple studies have revealed a consistent, though not universal, gut microbial signature in psoriasis patients: reduced alpha-diversity (a measure of community richness and evenness), an elevated *Firmicutes/Bacteroidetes* ratio, specific depletion of SCFA-producing genera (*Faecalibacterium prausnitzii*, *Roseburia* spp.), and an increase in pro-inflammatory species (13, 40, 41). The functional consequences of this ecological shift are profound and multi-layered:

#### 2.4.2 Barrier breakdown

A healthy gut microbiota supports intestinal epithelial integrity. Dysbiosis disrupts the expression of tight junction proteins (Occludin, ZO-1), leading to a state of increased intestinal permeability, colloquially termed “leaky gut” (42). This breach allows microbial-associated molecular patterns (MAMPs) like lipopolysaccharide (LPS) from Gram-negative bacteria and bacterial DNA to translocate into the portal and systemic circulation. These molecules act as danger signals, triggering innate immune activation via pattern recognition receptors (TLR4) on circulating monocytes and endothelial cells, resulting in low-grade systemic inflammation characterized by elevated CRP, IL-6, and TNF-α (42, 43).

#### 2.4.3 Metabolite-driven immunomodulation

The depletion of SCFA producers is arguably one of the most significant functional deficits. SCFAs (butyrate, propionate, acetate) are crucial anti-inflammatory metabolites produced by bacterial fermentation of dietary fiber. Butyrate is the primary energy source for colonocytes and is essential for maintaining barrier function. Systemically, SCFAs, particularly butyrate, promote the differentiation and function of regulatory T cells (Tregs) in the gut and periphery, crucial for maintaining immune tolerance (44). They also act as histone deacetylase (HDAC) inhibitors, leading to an anti-inflammatory chromatin state in immune cells, which suppresses the expression of pro-inflammatory genes like *IL6* and *IL12b* (45). Their reduction in psoriasis tips the systemic immune balance away from tolerance and towards inflammation, effectively priming the immune system for Th17 responses and reducing the braking capacity of Tregs.

#### 2.4.4 Direct immune cell interaction

Certain gut commensals can directly shape the host immune repertoire. The canonical example is segmented filamentous bacteria (SFB), which are potent inducers of Th17 cells in the murine small intestine lamina propria (46). These gut-derived Th17 cells can potentially migrate to extra-intestinal sites like the skin. While SFB has not been consistently identified in human studies, other bacteria like *Bifidobacterium adolescentis* have been shown to induce Th17 cells in human mononuclear cell cultures (47). This exemplifies a direct microbial mechanism for driving the key pathogenic cell type in psoriasis.

#### 2.4.5 The gut-skin axis

The systemic inflammation and activated immune cells (Th17 cells, γδ T cells) generated in the gut-associated lymphoid tissue (GALT) can traffic to the skin, where they participate in lesion formation. This is facilitated by the upregulation of skin-homing receptors (e.g., cutaneous lymphocyte antigen, CLA) on immune cells conditioned by gut dendritic cells (48). Furthermore, circulating microbial metabolites (like depleted SCFAs or increased deleterious metabolites) and inflammatory mediators (like LPS-induced cytokines) can directly affect cutaneous immunity and keratinocyte function. For instance, systemically low SCFA levels may reduce Treg suppression in the skin, while circulating LPS can prime dermal macrophages. This establishes a continuous dialogue where gut dysbiosis sustains a pro-inflammatory, Th17-permissive milieu that fuels and potentially initiates skin inflammation, even in the absence of overt gut symptoms.

### 2.5 Cutaneous microbiome perturbations

The skin of psoriatic lesions is not a sterile inflammatory field but hosts its own distinct dysbiotic ecosystem: increased overall bacterial load, reduced diversity, and a specific imbalance where *Staphylococcus* (especially *S. aureus*) and *Corynebacterium* are enriched, and beneficial commensals like *Cutibacterium* (formerly *Propionibacterium*) are diminished (49–51). This is not a passive reflection of the altered skin environment (e.g., increased moisture, scale) but an active contributor to pathogenesis:

#### 2.5.1 Barrier disruption and antigen presentation

*S. aureus* is a notorious pathobiont in psoriasis. It can produce proteases (V8 protease) that degrade corneodesmosomes, critical structures for epidermal cohesion, thereby physically impairing barrier function (52). Its antigens (e.g., staphylococcal enterotoxins that act as superantigens) can be presented by Langerhans cells and other cutaneous antigen-presenting cells, driving potent, polyclonal localized T cell responses that can include IL-17-producing cells (53).

#### 2.5.2 Direct immune activation

Microbial components from the dysbiotic community, such as lipoteichoic acid (LTA) from Gram-positive bacteria or peptidoglycan, can activate keratinocytes and resident immune cells via pattern recognition receptors (TLRs 2/6, NOD2). This activation leads to the production of IL-1β, IL-6, IL-23, and TNF-α from the very first stages, thereby initiating and perpetuating the inflammatory cascade from within the skin microenvironment (34, 54). This creates a feedback loop: inflammation alters the skin milieu, favoring *Staphylococcus* over *Cutibacterium*, which in turn produces more pro-inflammatory signals.

#### 2.5.3 Fungal involvement

Increased colonization with *Candida* spp., particularly in intertriginous areas, is common in psoriasis patients (55, 56). *Candida* is a potent natural inducer of Th17 responses, as antifungal immunity heavily relies on this axis. Anti-*Candida* immune responses (elevated serum IgA) are correlated with psoriasis severity and a Th17-dominant phenotype, suggesting a pathogenic role for the mycobiome beyond simple colonization (57). Fungal dysbiosis may be especially relevant in the context of biologic therapies that target IL-17, which are associated with increased candidiasis risk (58).

### 2.6 Metabolic comorbidities: the inflammatory nexus

Obesity, metabolic syndrome, and insulin resistance are common comorbidities that exacerbate psoriasis severity and reduce treatment response (59). This link is mechanistically robust. Adipose tissue, especially visceral fat, is not inert storage but an active endocrine organ secreting pro-inflammatory cytokines (TNF-α, IL-6) and adipokines like leptin (pro-inflammatory) while reducing adiponectin (anti-inflammatory) (60). This creates a state of “meta-inflammation.” The improvement of psoriasis with GLP-1 receptor agonists like semaglutide in obese patients, independent of significant weight loss in short-term studies, demonstrates a direct metabolic-immune-skin link (61). Dysbiosis is a key contributor to metabolic dysfunction. A diet high in fat and sugar can promote gut dysbiosis, which in turn increases intestinal permeability and systemic LPS levels (metabolic endotoxemia), driving insulin resistance and adipose tissue inflammation (62). This creates a vicious cycle: poor diet → gut dysbiosis/metabolic syndrome → systemic inflammation → worsened psoriasis → potential reduction in physical activity → further metabolic decline. Breaking this cycle through dietary intervention, weight loss, or metabolically active drugs may have direct benefits for skin disease **Table 1**.

**Table 1:** Functional consequences of microbiome dysbiosis in psoriasis.

Site of dysbiosis	Key taxonomic changes	Proposed functional consequences & mechanisms	Impact on psoriasis pathogenesis	References
Gut Microbiome	↓ Alpha-diversity ↓ *Bacteroidetes* ↓ SCFA-producers (e.g., *Faecalibacterium*, *Roseburia*) ↑ *Firmicutes/Bacteroidetes* ratio	*1.Impaired Barrier (“Leaky Gut”):* Dysbiosis reduces expression of tight junction proteins (e.g., Occludin, ZO-1), increasing intestinal permeability. *2. Reduced Immunoregulation*: Depletion of SCFAs (butyrate, propionate) diminishes Treg induction and anti-inflammatory HDAC inhibition. *3. Systemic Immune Activation:* Translocation of MAMPs (e.g., LPS) triggers innate immunity (via TLR4) and low-grade systemic inflammation (↑ CRP, IL-6, TNF-α). *4. Metabolic Shift*: Altered microbial bile acid and metabolite profiles influence host metabolism and inflammation.	Creates a pro-inflammatory systemic milieu that primes for Th17 responses. Compromised gut barrier allows microbial antigens and inflammatory mediators to enter circulation, which can traffic to and activate the skin. The loss of SCFA-mediated tolerance reduces systemic and cutaneous Treg function, lowering the threshold for psoriatic plaque formation and exacerbation.	(13, 40–45, 62)
Skin Microbiome	↓ Diversity ↑ *Staphylococcus* (esp. *S. aureus*) ↑ *Corynebacterium* ↓ *Cutibacterium*	*1.Barrier Disruption*: Bacterial proteases (e.g., V8 protease from *S. aureus*) degrade corneodesmosomes, impairing epidermal cohesion *2.Direct Pro-Inflammatory Stimulation:* MAMPs (e.g., LTA, peptidoglycan) activate TLRs/NLRs on keratinocytes and immune cells, inducing IL-1β, IL-6, IL-23, and TNF-α. *3.Antigenic Drive*: Bacterial antigens (e.g., staphylococcal enterotoxins) act as superantigens, driving polyclonal T cell activation, including IL-17-producing cells. *4.Loss of Beneficial Signals:* Depletion of commensals like *Cutibacterium* reduces beneficial metabolites (e.g., SCFAs) that support skin barrier integrity and hydration.	Drives a local self-sustaining inflammatory loop within psoriatic lesions. Pathobiont dominance directly triggers and amplifies the IL-23/Th17 axis from within the skin microenvironment. Barrier breakdown facilitates further microbial colonization and antigen exposure, while loss of commensal homeostasis removes natural anti-inflammatory signals.	(34, 49–54)
Oral Microbiome	↓ *Neisseria* (specifically noted in palmoplantar pustulosis)	Alters local immune surveillance; may promote systemic inflammation through bacterial translocation or molecular mimicry between oral microbial and self-antigens.	May contribute to specific clinical subtypes like palmoplantar pustulosis. The oral cavity serves as a potential reservoir for systemic immune activation, with dysbiosis possibly linking oral health to cutaneous disease severity via shared inflammatory pathways.	(63)
Fungal Community (Mycobiome)	↑ *Candida* colonization (skin & gut)	*Activation of Th17 Responses: Candida* is a potent natural inducer of Th17 immunity. Fungal antigens drive a robust anti-fungal response that overlaps with the psoriatic inflammatory pathway.	Provides a chronic antigenic stimulus that fuels the pathogenic IL-23/Th17 axis. Elevated anti-*Candida* IgA correlates with disease severity, indicating a pathogenic role beyond colonization. This interaction also explains the increased risk of candidiasis in patients on anti-IL-17 therapies.	(55–58)

## 3 Therapeutic mechanisms, biological insights, and the microbiome interface

Treatment effects provide compelling reverse-engineering evidence for the microbiome’s role. Therapies not only correct immune dysregulation but also induce shifts in microbial ecosystems, suggesting part of their efficacy may be microbiome mediated. This section explores these bidirectional interactions in detail, examining how different drug classes impact the microbiome and how microbial composition might influence therapeutic outcomes.

### 3.1 Biologics: targeted cytokine inhibition and microbial shifts

Biologics induce drug-specific microbiome alterations, revealing a fascinating bidirectional relationship where correcting host immunity reshapes the microbial landscape, and vice versa.

#### 3.1.1 Anti-IL-17A (Secukinumab/Ixekizumab)

These highly effective agents profoundly reshape the gut microbiome. Studies show they significantly increase the phylum *Proteobacteria* and decrease the *Firmicutes/Bacteroidetes* ratio; a shift distinct from both baseline psoriasis and healthy controls (64). The clinical significance of this specific change (increased *Proteobacteria*, which includes many Gram-negative genera) requires careful investigation. It may represent a beneficial reduction in inflammation-associated *Firmicutes* clusters, or it could be a potentially dysbiotic shift associated with treatment, though not causing adverse effects in most patients. Their potent suppression of IL-17 also significantly impacts the skin mycobiome, increasing the risk of mucocutaneous candidiasis, directly demonstrating the role of IL-17 in controlling *Candida* (58).

#### 3.1.2 Anti-IL-23p19 (Risankizumab, Guselkumab, Tildrakizumab)

These agents target the upstream cytokine responsible for Th17 cell maintenance. They show superior PASI 90 response rates compared to other therapies like apremilast, and even switching non-responders from other biologics to anti-IL-23 agents can yield high efficacy (65). Their impact on the microbiome is less studied but is theoretically profound. By interrupting the IL-23-dependent maintenance of not only circulating Th17 cells but also tissue-resident memory T cells (TRM) in the gut and skin, they may allow for a more fundamental “resetting” of the pathogenic immune niche, potentially leading to more durable microbial normalization (66).

#### 3.1.3 Anti-TNF-α (Adalimumab, Infliximab, Etanercept)

TNF-α inhibitors are effective for psoriasis and psoriatic arthritis. Response has been correlated with a molecular index of epidermal pathology (MITE score), linking clinical improvement to keratinocyte normalization (28). However, TNF-α is a pleiotropic cytokine crucial for host defense, particularly in granuloma formation. Thus, its inhibition is associated with an increased risk of certain infections, including reactivation of latent tuberculosis (TB), invasive fungal infections, and hepatitis B virus (HBV) reactivation (67–69). This highlights the delicate balance between suppressing pathological immunity and compromising essential host defense pathways, a balance that can be influenced by the patient’s microbial and viromic carriage.

#### 3.1.4 Ustekinumab (anti-IL-12/23p40)

This agent blocks both IL-12 (Th1-related) and IL-23 (Th17-related). Longitudinal studies show it modulates the skin microbiome by increasing site-specific community heterogeneity and beta-diversity over time, suggesting a normalization towards a healthier, more diverse and site-appropriate state as inflammation resolves (70). This effect might be due to the broad suppression of multiple inflammatory axes that shape the cutaneous environment.

### 3.2 Small molecules and microbiome-targeted interventions

#### 3.2.1 PDE4 inhibitors (apremilast)

This oral small molecule increases intracellular cAMP levels in immune cells, leading to downregulation of pro-inflammatory cytokines (TNF-α, IL-17, IL-23) and upregulation of anti-inflammatory mediators (IL-10). It is effective for moderate plaque psoriasis and particularly for nail psoriasis (71). Its oral administration and anti-inflammatory effects likely influence the gut microbiome, though detailed studies are needed.

#### 3.2.2 Probiotics & synbiotics

Direct intervention in the gut microbiome provides proof-of-concept for its role. Specific probiotic mixtures (e.g., containing *Lactobacillus* and *Bifidobacterium* strains) have shown promise in randomized controlled trials (RCTs), improving PASI scores, reducing relapse rates after conventional therapy, and even enhancing mineral absorption (with the synbiotic Lactocare^®^) (72, 73). These benefits are postulated to occur via restoration of barrier function, increase in SCFA production, direct immune modulation, and reduction of systemic inflammatory tone.

#### 3.2.3 Fecal microbiota transplantation

In contrast to targeted probiotics, FMT aims to engraft an entire healthy donor ecosystem. However, an RCT in active peripheral psoriatic arthritis found FMT ineffective and inferior to sham treatment (74). This negative result is crucial. It underscores the immense complexity of microbial therapeutics; factors like donor selection (lack of a defined “super-donor” for psoriasis), route of administration, insufficient engraftment in the context of ongoing joint inflammation, and patient stratification (baseline dysbiosis profile) are likely critical for success. It suggests that simply transferring a “healthy” microbiome may not be sufficient to override established inflammatory circuits in musculoskeletal tissues.

### 3.3 Summary of key clinical evidence

The key findings from pivotal clinical studies investigating various therapeutic, microbiome-targeted, and mechanistic interventions in psoriasis are summarized in **Table 2**. This evidence underpins the discussions on therapeutic mechanisms and the microbiome’s role, highlighting both promising avenues and negative results that shape current understanding.

**Table 2:** Summary of clinical studies on psoriasis.

Aim of the study	Main findings	Conclusion	Reference
Examine calcipotriol effect on immune cells in psoriasis lesions.	↓ Frequency of CD8+ IL-17+ T cells (p<0.05). No change in CD4+, ILCs, IL-22+, or IFN-γ+ cells.	Vitamin D analog reduces IL-17-producing CD8+ T cells in lesions, contributing to clinical improvement.	(75)
Validate Molecular Index of Therapeutic Efficacy (MITE) for monitoring anti-TNF-α response.	MITE score (epidermal thickness/Ki67/K17/CRABP-II) correlated with PASI/PDI (p<0.001). ↓ MITE in responders.	MITE score reliably monitors clinical and biomolecular treatment response in psoriasis.	(28)
Compare efficacy/safety of risankizumab (anti-IL-23) vs. apremilast (PDE4i).	Risankizumab superior: PASI90 55.9% vs. 5.1% at wk16. Switched non-responders: PASI90 72.3% vs. 2.6% at wk52.	IL-23 inhibition significantly outperforms PDE4 inhibition. Switching non-responders to risankizumab improves outcomes.	(65)
Explore IκBζ role in secukinumab’s (anti-IL-17A) mechanism.	IκBζ ↓ by day 4, correlating with PASI. Regulates psoriasis signature genes via Act1/p38/JNK/NF-κB.	IκBζ is a key early mediator and central node in the antipsoriatic effects of IL-17A blockade.	(26)
Evaluate semaglutide (GLP-1 RA) effect on psoriasis in obese T2DM patients.	PASI significantly improved (21→10; p=0.002). IL-6/CRP decreased. BMI/LDL reduced. Quality of life enhanced.	Highlights metabolic-immune-skin link. Suggests GLP-1 RAs as a therapeutic strategy for psoriasis with metabolic comorbidity.	(61)
Assess efficacy/safety of a specific probiotic mixture in plaque psoriasis.	66.7% probiotic vs. 41.9% placebo achieved PASI75 (p<0.05). Lower relapse risk at 6 months. Gut microbiota modulation confirmed.	Provides direct proof-of-concept that modulating gut microbiota can reduce psoriasis severity and relapse.	(73)
Evaluate Lactocare^®^ synbiotic effect on serum trace elements.	↑ Fe, Zn, P, Mg, Ca, Na post-treatment (p<0.05). Sex influenced Fe/Cu levels.	Synbiotics improve mineral absorption (especially Fe, Zn, Ca) in psoriatic patients, suggesting a novel ancillary benefit.	(72)
Assess safety/efficacy of FMT vs. sham in active peripheral psoriatic arthritis (PsA).	FMT inferior: 60% vs. 19% treatment failure (p=0.018). HAQ-DI worsened. No serious adverse events.	FMT is safe but ineffective for active PsA vs. sham, underscoring complexity of microbial therapeutics.	(74)
Analyze skin microbiome dynamics during ustekinumab (anti-IL-12/23) therapy.	Minor site-specific diversity differences. Microbiota heterogeneity ↑ post-treatment. No specific psoriasis taxa identified.	Treatment increases microbial community variance, reflecting a restoration of healthy skin site specificity rather than a targeted shift.	(70)
Examine effect of high-dose vitamin D supplementation on psoriasis severity.	No significant PASI difference vs. placebo. Vitamin D levels increased less than expected. No adverse effects.	Vitamin D supplementation does not improve psoriasis severity in deficient patients, suggesting deficiency is not a primary driver.	(76)
Investigate relapse rates after secukinumab withdrawal.	20.8% (300mg) and 14% (150mg) relapse-free at 1 year. Shorter disease duration/lower baseline PASI linked to longer remission.	A subset achieves sustained remission after therapy cessation; early intervention may prolong treatment-free intervals.	(77)
Determine MTX effect on TB test (TST/IGRA) results.	TST conversions ↑ (25%→44%; p<0.037). Serum IFN-γ ↑ (p<0.005).	MTX may cause TST “boosting” via immune modulation (increased IFN-γ), complicating TB screening in psoriasis.	(78)
Test Huaier (TCM) efficacy in mild-moderate psoriasis.	PASI50/75/90 ↑ vs. placebo (p<0.01). Inhibited HaCaT proliferation, induced G1 cell cycle arrest *in vitro*.	Demonstrates clinical efficacy and direct antiproliferative effects on keratinocytes, highlighting potential of non-biologic agents.	(79)
Characterize immune response against *Candida albicans* in psoriasis.	Anti-CA IgA↑ in plaque psoriasis. Linked to CLA+ Th17 response. High IgA associated with severe disease biomarkers (CCL18, CHI3L1).	Anti-Candida IgA levels correlate with chronic psoriasis severity and Th17 activation, implicating the mycobiome in disease activity.	(57)

### 3.4 Relapse and remission: clues to sustained control

**Figure 2:**
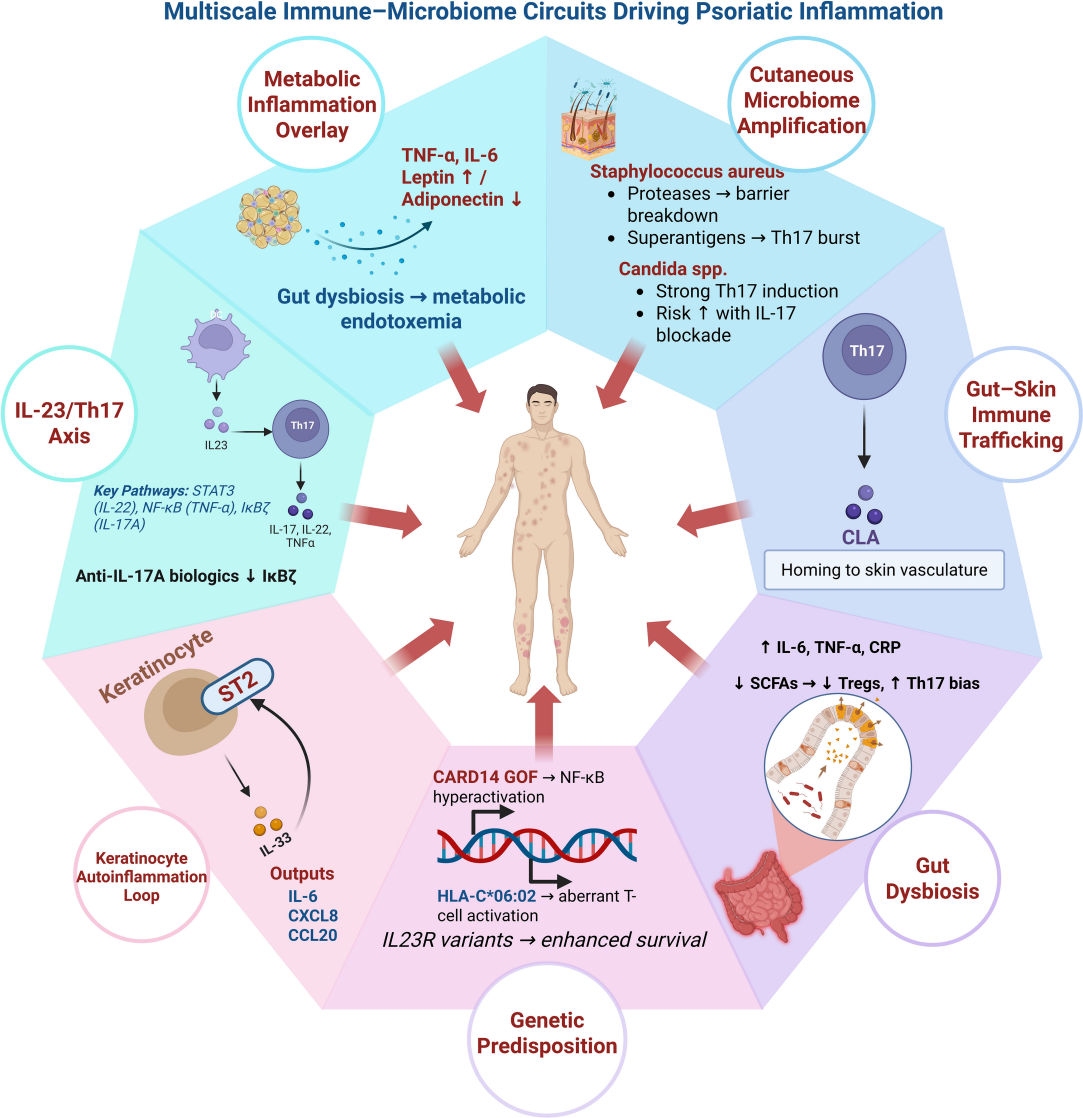
Multiscale immune–microbiome circuits driving psoriatic inflammation. Psoriasis is sustained by interconnected immune, epithelial, microbial, genetic, and metabolic pathways converging on chronic skin inflammation. Central to disease pathogenesis is the IL-23/Th17 axis, in which IL-23–driven Th17 cells produce IL-17A/F, IL-22, and TNF-α, activating keratinocyte STAT3/NF-κB signaling and the transcriptional regulator IκBζ. Keratinocytes further amplify inflammation through an intrinsic IL-33/ST2 autoinflammatory loop. Genetic susceptibility (e.g., HLA-C*06:02, CARD14 gain-of-function, IL23R variants) lowers immune activation thresholds. Gut dysbiosis promotes systemic inflammation and Th17 bias via barrier disruption, reduced short-chain fatty acids, and metabolic endotoxemia, enabling immune cell trafficking to the skin. Cutaneous microbiome perturbations and metabolic inflammation further reinforce this self-amplifying inflammatory network.

The study of what happens after therapy cessation provides unique insights into disease mechanisms and the potential for sustained immune reset. Following secukinumab withdrawal, a subset of patients (approximately 15-20%) remains relapse-free for a year or more, an outcome associated with shorter disease duration and lower baseline severity (77, 78). This suggests that early, potent intervention may reset immune homeostasis in some individuals, possibly by depleting long-lived pathogenic tissue-resident memory T cells (TRM) or by inducing a more tolerant immune microenvironment. The microbiome composition pre- and post-treatment could be a key predictor of such sustained remission. Patients who achieve a “healthier,” more diverse microbial profile during treatment might be more likely to maintain remission, as this restored ecosystem could support ongoing immune regulation. Conversely, rapid return to a dysbiotic state after drug cessation might precipitate relapse. Investigating microbial and metabolomic biomarkers associated with treatment-free remission is a critical future direction **Figure 2**.

## 4 Limitations of current research and the causality gap

Despite compelling associations, significant limitations impede translational progress from observational links to causative understanding and clinical application. These constraints are multi-faceted and interdependent.

### 4.1 Methodological heterogeneity

The lack of standardized protocols across studies creates noise that can drown out true biological signals. For instance, skin swabs may capture surface microbes while biopsies include the follicle microbiome, representing distinct niches (80). This inconsistency prevents the identification of a universal “core” psoriatic dysbiosis and reliable biomarkers (81).

### 4.2 The causality conundrum

Observational, cross-sectional studies can only establish association. The gold standard for causality; randomized intervention, is challenging in microbiome research. While Mendelian Randomization studies using genetic instruments offer some evidence for a potential causal role of gut microbiota in psoriasis (4), they have limitations. Animal models, though valuable for mechanistic insight, do not fully recapitulate human psoriasis complexity, and human FMT trials have so far been disappointing for psoriatic arthritis (74, 82).

### 4.3 Functional black box

Knowing which microbes are present is only the first step. The critical link between microbial shifts and host physiology remains opaque. We lack understanding of the functional output of the dysbiotic community: What metabolites are being produced or depleted? Which microbial genes are being expressed? How does this alter host cell gene expression and signaling in the gut and skin? Integrating metagenomics (for functional potential), metatranscriptomics (for active functions), metabolomics (for microbial and host metabolites), and host immunophenotyping in the same subjects is essential but costly and complex (83).

### 4.4 Narrow taxonomic focus

The research lens has been overwhelmingly trained on bacteria. The fungal community (mycobiome), particularly *Malassezia* on the skin and *Candida* in the gut, is implicated in psoriasis and interacts with bacteria and the immune system (55, 56). The virome (bacteriophages and eukaryotic viruses) can shape bacterial community structure through predation and can directly modulate host immunity, but it is rarely studied (20). This bacterial-centric view provides an incomplete picture of the microbial ecosystem.

### 4.5 Inadequate control of confounders

The microbiome is highly personalized and influenced by a myriad of factors. Studies that fail to rigorously account for medication history (especially recent antibiotics), diet, and lifestyle risk attributing differences to psoriasis that are actually due to these confounders. This obscures the true disease-specific microbial signature and can lead to spurious associations (19).

## 5 Prospective research directions: from association to mechanism and application

Future research must address the above limitations with targeted, integrated strategies to move the field from descriptive ecology to mechanistic microbiology with clinical utility.

### 5.1 Functional multi-omics studies

Future studies must adopt a holistic, functional approach. Combining shotgun metagenomics (to assess the genetic potential of the microbiome), metatranscriptomics (to see which genes are actively expressed under disease conditions), metabolomics (lipidomics, etc., to measure the resulting metabolite flux), and host single-cell RNA sequencing of skin and blood immune cells in longitudinal samples will reveal the active functional dialogue between host and microbes (83). This can identify keystone functions, not just keystone taxa, that drive pathology.

### 5.2 Resolving causality

Prospective cohort studies starting in pre-disease states (first-degree relatives) are essential. Well-designed, mechanistic interventional trials are needed: FMT trials with careful donor-recipient matching, perhaps using donors who have themselves achieved deep remission on therapy; trials of targeted antimicrobials against pathobionts like *S. aureus* combined with probiotics; and dietary interventions (high-fiber, prebiotic diets) with multi-omics readouts (47). Sophisticated humanized mouse models will continue to be vital for testing causality of specific microbial strains or functions.

### 5.3 Exploring understudied pathways

#### 5.3.1 Targeting keratinocyte loops

Clinical trials of anti-IL-33/ST2 biologics (tozorakimab) for moderate-to-severe psoriasis are underway and will test the importance of this keratinocyte-intrinsic amplifier (33).

#### 5.3.2 Intracellular targets

IκBζ is a promising intracellular target. Developing specific, safe small-molecule inhibitors of IκBζ or its upstream activators could provide a novel oral therapeutic strategy that blocks a central convergence point for multiple inflammatory signals (24).

#### 5.3.3 Metabolic comorbidity axis

Large, dedicated RCTs of GLP-1 receptor agonists, SGLT2 inhibitors, and other metabolically active drugs for psoriasis in patients with obesity/metabolic syndrome, independent of diabetes status, are needed to firmly establish this treatment axis (61).

### 5.4 Personalized biomarker development

Research should focus on validating microbial signatures (e.g., a specific gut profile predicting anti-IL-17 response), serum or fecal metabolomic profiles (low butyrate levels), and genetic markers (HLA-C06:02) to create composite predictive algorithms. These tools could guide treatment selection (biologic choice), identify patients at high risk for PsA, or predict who might achieve sustained remission after therapy cessation (21).

### 5.5 Long-term safety ecosystems

**Figure 3:**
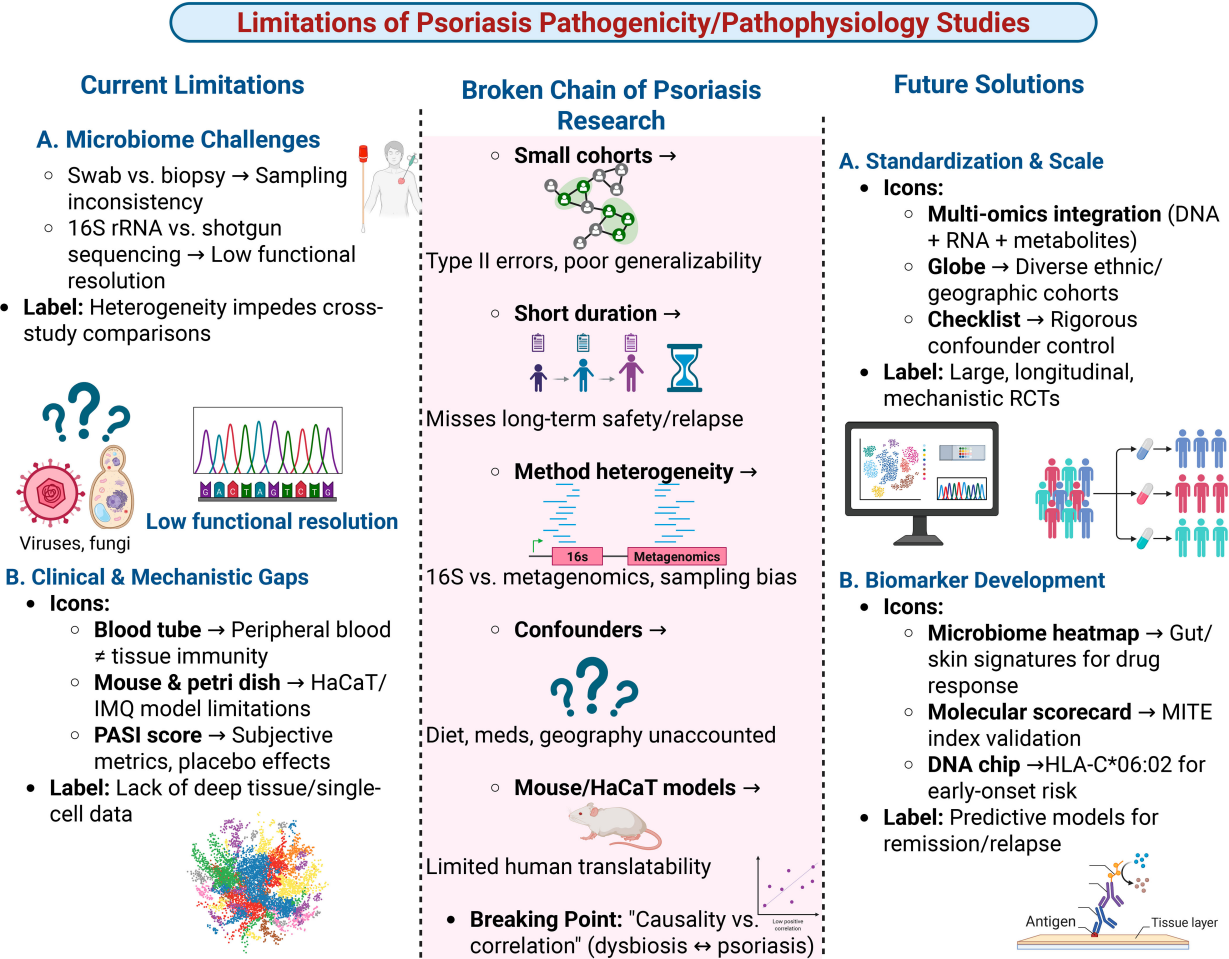
Current limitations and future directions in psoriasis microbiome research. The schematic summarizes key methodological and conceptual gaps that have limited causal inference in psoriasis microbiome studies. Current challenges include sampling inconsistency, low functional resolution of sequencing approaches, small and short-duration cohorts, methodological heterogeneity, inadequate control of confounders, and limited translatability of animal and in vitro models. These factors contribute to a fragmented research chain and hinder robust generalization. Proposed future solutions emphasize standardized, large-scale, longitudinal multi-omics studies across diverse populations, rigorous confounder control, and integration of microbiome-derived biomarkers. Such approaches aim to enable mechanistic clarity, improve therapeutic stratification, and support predictive models of treatment response, remission, and relapse.

As treatment options expand, understanding their long-term impact is crucial. Prospective registries should systematically collect data on infections (including opportunistic and reactivated latent infections), cardiovascular events, metabolic parameters, and malignancy incidence in patients on newer biologics and microbiome-targeted therapies (83, 84). This will enable better risk stratification and management strategies **Figure 3**.

## 6 Conclusion

This review advances the understanding of psoriasis by framing microbiome dysbiosis as a central mechanistic player within the established genetic-immune paradigm. We have detailed a model where gut dysbiosis erodes systemic immune tolerance and promotes a Th17-biased inflammatory milieu, while skin dysbiosis provides local triggers and amplifiers, together forming a self-reinforcing “Gut-Skin-Inflammatory Axis.” Specific infections can exploit this dysregulated system. The bidirectional interaction with therapies; where effective treatment often normalizes microbial communities, further solidifies this link.

However, the field is at a crossroads, constrained by a causality gap and methodological inconsistencies. The promise of microbiome-based diagnostics and therapeutics is real, as evidenced by successful probiotic trials, but it remains unfulfilled. The path forward requires a concerted shift from descriptive ecology to mechanistic microbiology. This entails large-scale, longitudinal, multi-omics studies that can capture the dynamic interplay between host and microbes, coupled with innovative interventional trials targeting specific microbial functions or host-microbe interaction points. By embracing this complexity, we can move towards a future of personalized management for psoriasis, where modulating the microbiome becomes a strategic component of inducing deep, sustained remission and addressing the root causes of this chronic disease.
